# Association of depression symptoms and sleep quality with state-trait anxiety in medical university students in Anhui Province, China: a mediation analysis

**DOI:** 10.1186/s12909-022-03683-2

**Published:** 2022-08-19

**Authors:** Jiangyun Chen, Yusupujiang Tuersun, Jiao Yang, Man Xiong, Yueying Wang, Xinyi Rao, Shuai Jiang

**Affiliations:** 1 School of Health Management, Southern Medical University, Guangzhou, China; 2 The First Affiliated Hospital of Zhengzhou University, Zhengzhou, China; 3 Institute for Hospital Management of Henan Province, Zhengzhou, China

**Keywords:** Students, Medical, Sleep quality, Depression symptoms, Anxiety, China

## Abstract

**Background:**

The prevalence of depression symptoms among medical students is particularly high, and it has increased during the COVID-19 epidemic. Sleep quality and state-trait anxiety are risk factors for depression, but no study has yet investigated the mediating role of state-trait anxiety in the relationship between poor sleep quality and depression symptoms in medical students. This study aims to investigate the relationship among depression symptoms, sleep quality and state-trait anxiety in medical university students in Anhui Province.

**Methods:**

This was a cross-sectional survey of 1227 students’ online questionnaires collected from four medical universities in Anhui Province using a convenience sampling method. We measured respondents’ sleep quality, state-trait anxiety, and depression symptoms using three scales: the Pittsburgh Sleep Quality Index (PSQI), the State-Trait Anxiety Inventory (STAI) and the Self-rating Depression Scale (SDS). We analysed the mediating role of STAI scores on the association between PSQI scores and SDS scores through the Sobel-Goodman Mediation Test while controlling for covariates. *P* < 0.05 was considered statistically significant.

**Results:**

A total of 74.33% (912) and 41.40% (518) of the respondents reported suffering from poor sleep quality and depression symptoms. Sleep quality, state-trait anxiety, and depression symptoms were positively associated with each other (β = 0.381 ~ 0.775, *P* < 0.001). State-trait anxiety partially mediated the association between sleep quality and depression symptoms (Sobel test Z = 15.090, *P* < 0.001), and this mediating variable accounted for 83.79% of the association when adjusting for potential confounders. Subgroup analysis further revealed that STAI scores partially mediated the association between PSQI scores and SDS scores in females and rural students and fully mediated the association between PSQI scores and SDS scores in males and urban students.

**Conclusions:**

This study found that sleep quality and state-trait anxiety have a significant predictive effect on depression symptoms. State-trait anxiety mediated the relationship between sleep quality and depression symptoms, with a more complex mechanism observed among rural and female medical students. Multiple pathways of intervention should be adopted, such as encouraging students to self-adjust, providing professional psychological intervention and timely monitoring, enriching extracurricular activities, and making changes in policies regarding long shifts and working hours.

**Supplementary Information:**

The online version contains supplementary material available at 10.1186/s12909-022-03683-2.

## Background

According to the World Health Organization, 264 million people of all ages worldwide suffered from depression symptoms in 2017, among which the lifetime prevalence of depression symptoms in China was 6.9%, and the proportion of Chinese undergraduates suffering from depression symptoms was as high as 23.7% [1].

Several studies have shown that medical students have a higher prevalence of depression than nonmedical students due to academic stress [2–4]. Additionally, the prevalence of depression symptoms among medical students has increased during the COVID-19 epidemic [5–7]. According to Scott J Halperin, the prevalence of depression symptoms was 70% higher in the COVID-19 era than in previous studies of medical students [5]. A study by Franck Rolland showed a higher prevalence of 12-month major depressive episodes among medical students one year after the start of the COVID-19 pandemic than in 2016 [6]. Yoshito Nishimura also reported that online education during the COVID-19 pandemic aggravated the depression symptoms of medical students [7]. Depression symptoms among medical students can lead to many negative consequences. First, the development of suicidal ideation caused by depression symptoms in medical students is alarming, with studies showing that the incidence of depression or depression symptoms was 28.0% and that the incidence of suicidal ideation among medical students was 5.8% [8]. Second, depressive symptoms have a negative impact on the academic performance of medical students [3]. Third, their professional careers and personal lives will suffer negative effects if depression symptoms are not treated [9]. Fourth, students with depressive symptoms exhibit relationship problems, cynicism, and a decline in satisfaction with academic activities [10]. Clearly, there is a need to pay attention to the depression symptoms of medical students.

Medical students’ sleep quality is poor due to late-night studying [11, 12], a bad dormitory environment [13], excessive use of electronic devices before bedtime [14, 15], and internship shifts [16]. The study showed that the prevalence of sleep disturbance among medical students is higher than that among nonmedical students and the general population [17]. Additionally, sleep disturbance is a risk factor for depression symptoms [18]. The occurrence of depression symptoms was related to lower sleep quality [11, 19].

In addition, the state-trait anxiety status of medical students is strongly associated with depression symptoms. Depression symptoms and anxiety disorders coexist almost simultaneously, and an article examining factors associated with depression symptoms and anxiety in medical students showed a positive correlation between levels of state anxiety and trait anxiety and depression scores [20]. It has been shown that anxiety precedes depression episodes for most older age groups [21]. A previous study identified anxiety as a predictor of depression symptoms in medical students [22]. High levels of state-trait anxiety were a cause of medical students maintaining a depressive state [10].

Sleep quality affects the occurrence of state-trait anxiety. A previous survey showed that people with poor sleep reported a higher frequency of anxiety [23]. A 9-year follow-up survey showed that sleep difficulties significantly predicted self-reported anxiety six to nine years later, and as self-reported sleeping difficulty severity increased, the risk of depression also increased [24].

In summary, it has been proven that poor sleep quality can lead to depression symptoms; that higher levels of state-trait anxiety are risk factors for depression symptoms; and that poor sleep quality can cause state-trait anxiety. However, the relationship between the three is still unclear. Additionally, depression symptoms among medical students worsened due to the outbreak of COVID-19, which affected their academic, health, and interpersonal relationships. It is important to understand the condition and the mechanisms associated with the occurrence of depression symptoms in medical students in the context of COVID-19.

### Present study

In this study, data on sleep quality, state-trait anxiety and depression symptoms among medical students in four medical universities in Anhui Province were collected using scales. The aim of this study was to investigate the mediating role of state-trait anxiety in the relationship between sleep quality and depression symptoms. Based on a literature review, the following hypotheses were formulated: quality of sleep directly affects depression symptoms and indirectly affects depression symptoms through state-trait anxiety; that is, state-trait anxiety mediates the relationship between quality of sleep and depression symptoms.

## Methods

### Subjects and procedure

A cross-sectional survey of university students from four medical universities in Anhui Province was conducted from September to December 2020 using a convenience sampling method. To better identify medical students, we included all medical universities in Anhui, leaving out comprehensive universities. We included in this experiment all medical universities in Anhui Province (there are four medical universities in total in Anhui Province). These universities offer undergraduate degree education, including Anhui Medical University, Anhui University of Traditional Chinese Medicine, Bengbu Medical College, and Wannan Medical College (the number of undergraduate students at each university ranges from 12,000 to 16,000). We distributed online questionnaires to university students with the assistance of academic administrators using the web-based Questionnaire Star platform (https://www.wjx.cn/) and obtained informed consent from each participating subject. In addition, because face-face surveys were difficult to conduct during the COVID-19 outbreak and university students were the main users of mobile devices, they could also complete the questionnaire accurately on their own. The questionnaire comprised four main sections. The first section obtained the sociodemographic characteristics of the participants (e.g., age, gender, grade). The second section assessed the level of sleep quality of the medical students. The third part assessed the level of state-trait anxiety of the medical students. In the fourth part, we assessed the level of depression symptoms of the medical students (Supplementary Table 1). The questionnaire was completed anonymously online, and subjects who provided electronic informed consent and voluntarily participated in the study were included in the study. The study protocol was approved by the Clinical Trials Ethics Committee of the First Affiliated Hospital of Zhengzhou University. The ethical approval number is 2021-KY-0669.

### Sleep quality (PSQI)

Sleep quality was assessed using the Pittsburgh Sleep Quality Index (PSQI) [25]. The index consists of 19 self-rated items across seven components on a scale from 0 to 3, where 0 indicates no difficulty and 3 indicates severe difficulty. The scores of these seven components are combined to provide a total score of 0–21, with higher scores indicating poorer sleep quality and scores greater than 5 distinguishing poor sleepers from good sleepers. The PSQI is considered an appropriate tool for assessing sleep quality in adults (18–80 years) [26], and Cronbach’s alpha among adolescents and young adults was 0.72 [27]. The PSQI in this study was divided into two levels of presence or absence of sleep disorder (1 = with sleep disorder, 2 = without sleep disorder) and four levels of degree of sleep quality (1 = fair sleep quality, 2 = fair sleep quality, 3 = very poor sleep quality, 4 = very good sleep quality) by the scores of the collected data. In the current sample, Cronbach’s alpha was 0.796, whereas the Kaiser–Meyer–Olkin (KMO) was 0.746. The significance of Bartlett’s test of sphericity was *P* < 0.05. The scale exhibited superior reliability and validity.

### State-Trait Anxiety Inventory (STAI)

The state-trait anxiety inventory (STAI) questionnaire consists of 40 questions divided into two groups to assess anxiety as a transient state (state anxiety) and as an underlying trait (trait anxiety). State anxiety is considered to be a transient emotional state characterized by subjective feelings, apprehension, and overactivity of the autonomic nervous system. Trait anxiety is a relatively stable personal state with a tendency to perceive situations as threatening. Both the state and trait scales consist of 20 items, including directly and inversely worded questions and punctuation. Scores range from 20 to 80, with higher scores indicating higher levels of anxiety [28, 29]. The final STAI scores were obtained using an online calculator (https://www.nsrusa.org/score.php) to avoid confusion about the punctuation of the reverse wording. The Cronbach’s α coefficient was 0.954, and the Kaiser–Meyer–Olkin (KMO) was 0.971. The significance of Bartlett’s test of sphericity was *P* < 0.05. The scale exhibited superior reliability and validity.

### Self-rating Depression Scale (SDS)

The Self-rating Depression Scale (SDS) is a brief self-rating scale that assesses the psychological and somatic symptoms of depression. It has been widely used in different age groups for screening purposes and to measure depression [30]. It has been widely used to screen for and measure the severity of depression [31]. It has good internal consistency test and retest reliability and has good content validity and criterion validity [32]. The standard converted score of the SDS is from 0 to 100 (raw score range from 20 to 80), and 50 and over suggests clinically significant symptoms [33]. The Cronbach’s α coefficient was 0.954, whereas the Kaiser–Meyer–Olkin (KMO) was 0.971. The significance of Bartlett’s test of sphericity was *P* < 0.05. The scale exhibited superior reliability and validity.

### Covariates

Age and sex were included as fixed covariates and were adjusted for in the analyses. Other covariates were included in the final model as potential confounders if they altered PSQI/STAI estimates of SDS by >10% or were significantly associated with SDS. The following covariates were selected based on established associations and/or plausible biological relationships: major, ethnicity, only child, birthplace, closest relationship, education of closest relationship, education of mother, education of father, job of closest relationship, job of mother, job of father. The relationship between each confounding factor and SDS is detailed in Supplementary Tables 2, 3 and 4.

### Statistical analysis

Continuous variables are reported as the mean ± SD, and categorical variables are reported as the frequency (%). Characteristic differences were examined using Student’s t test for continuous variables and the chi-squared test for categorical variables. Linear regression models were used to measure the association between PSQI and SDS/STAI scores before and after adjustment for covariates and the association between STAI and SDS scores and are reported as coefficients and 95% confidence intervals (CIs). We analysed the mediating role of STAI scores on the association of PSQI scores with SDS scores through the Sobel-Goodman Mediation Test [34] while controlling for all the selected covariates [35].

All *p* values were 2-sided, with an α ≤0.05 used to define statistical significance. Data were analysed using Stata version 16 (2017, University Station, Texas 77,845 USA) and R version 3.6.3 (2018, R Foundation for Statistical Computing, Vienna, Austria).

## Results

### General characteristics

A total of 1300 questionnaires were collected, with 1227 valid questionnaires (389 samples were obtained from Anhui Medical University, 288 samples from Anhui University of Traditional Chinese Medicine, 246 samples from Bengbu Medical University, and 304 samples from Wannan Medical University). Thus, the effective rate was 94.4%. Among the respondents, 594 were males, accounting for 48.4%, and 633 were females, accounting for 51.6%.

The demographics of the 1227 patients are presented in Table 1. The majority of students were in the first and fourth years, 23.5 and 55.1%, respectively, and 88.6% were majoring in medicine. A total of 64.8% of students were from rural areas, 43.5% of medical students’ fathers were workers, and 35.1% of their mothers were workers. Poor sleep quality was reported by 74.3% (912) of the respondents, and 41.4% (518) reported suffering from depression symptoms. There was a statistically significant difference in sleep quality scores between males and females, 27.3% (162) and 24.2% (153), respectively. State-trait anxiety and depression symptoms scores were nonsignificant in the distribution of males versus females (*P* > 0.05) (see Table 1 at the end of the article for details).

**Table 1:** Characteristics of respondents (*N* = 1227)

	Overall (*n* = 1227)	Male (*n* = 594)	Female (*n* = 633)	*P* value
Grade	N (%)			0.133
1	288 (23.47)	121 (20.37)	167 (26.38)	
2	75 (6.11)	39 (6.57)	36 (5.69)	
3	91 (7.42)	43 (7.24)	48 (7.58)	
4	676 (55.09)	345 (58.08)	331 (52.29)	
5	97 (7.91)	46 (7.74)	51 (8.06)	
Major				0.263
Medicine	1087 (88.59)	520 (87.54)	567 (89.57)	
Other	140 (11.41)	74 (12.46)	66 (10.43)	
Ethnicity				0.873
Han	1193 (97.23)	578 (97.31)	615 (97.16)	
Minority	24 (1.96)	16 (2.69)	18 (2.84)	
Only child				<0.001
No	740 (60.31)	314 (52.86)	426 (67.30)	
Yes	487 (36.69)	280 (47.14)	207 (32.70)	
Birthplace				0.225
Urban	432 (35.21)	199 (33.50)	233 (36.81)	
Rural	795 (64.79)	395 (66.50)	400 (63.19)	
Closet relationship				0.192
Parents	1006 (81.99)	492 (82.83)	514 (81.20)	
Grandparents	145 (11.82)	60 (10.10)	85 (13.43)	
Siblings	51 (4.16)	27 (4.55)	24 (3.79)	
Other	25 (2.04)	15 (2.53)	10 (1.58)	
Education of closest relationship				0.001
Less than lower secondary education	640 (52.16)	301 (50.67)	339 (52.97)	
Upper secondary & vocational	251 (20.46)	148 (24.92)	103 (16.27)	
Tertiary education	336 (27.38)	145 (24.41)	191 (30.17)	
Education of father				0.011
Less than lower secondary education	662 (53.95)	313 (52.69)	349 (55.13)	
Upper secondary & vocational	290 (23.63)	157 (26.43)	133 (21.10)	
Tertiary education	275 (22.41)	124 (20.88)	151 (23.85)	
Education of mother				0.010
Less than lower secondary education	820 (66.83)	386 (64.98)	434 (68.56)	
Upper secondary & vocational	235 (19.15)	136 (22.90)	99 (15.64)	
Tertiary education	172 (14.02)	72 (12.12)	100 (15.80)	
Job of closest relationship				0.003
Workers	396 (32.27)	226 (38.05)	170 (26.86)	
Farmers	206 (16.79)	94 (15.82)	112 (17.69)	
Civil servants, teachers and other intellectuals	264 (21.52)	110 (18.52)	154 (24.33)	
Businessmen	172 (14.02)	82 (13.80)	90 (14.22)	
Others	199 (16.22)	82 (13.80)	107 (16.90)	
Job of father				0.411
Workers	534 (43.52)	275 (46.30)	259 (40.92)	
Farmers	131 (10.68)	64 (10.77)	67 (10.58)	
Civil servants, teachers and other intellectuals	237 (19.32)	108 (18.18)	129 (20.38)	
Businessmen	197 (16.06)	94 (15.82)	103 (16.27)	
Others	128 (10.43)	53 (8.92)	75 (11.85)	
Job of mother				0.008
Workers	431 (35.13)	239 (40.24)	192 (30.33)	
Farmers	243 (19.80)	112 (18.86)	131 (20.70)	
Civil servants, teachers and other intellectuals	185 (15.08)	86 (14.48)	99 (15.64)	
Businessmen	154 (12.55)	72 (12.12)	82 (12.95)	
Others	214 (17.44)	85 (14.31)	129 (20.38)	
PSQI (Mean ± SD)	5.87 ± 2.94	5.75 ± 3.04	5.97 ± 2.86	0.010
STAI (Mean ± SD)	84.10 ± 17.28	84.23 ± 17.76	83.98 ± 16.82	0.278
SDS (Mean ± SD)	49.04 ± 10.85	48.87 ± 11.09	49.19 ± 10.62	0.228

### Correlation analysis

SDS scores were positively correlated with PSQI scores (*r* = 0.381, *p* < 0.001) and STAI scores (*r* = 0.775, *p* < 0.001), and PSQI scores were positively correlated with STAI scores (*r* = 0.428, *p* < 0.001) when controlling for confounding factors (Table 2).

**Table 2:** Partial correlations coefficients (r) among PSQI, STAI and SDS

	PSQI	STAI	SDS
PSQI			
STAI	0.428***		
SDS	0.381***	0.775***	

*PSQI *Pittsburgh Sleep Quality Index, *STAI *State-Trait Anxiety Inventory, *SDS *Self-rating Depression Scale

The model was adjusted for gender, birthplace, grade, major, ethnicity, only child, close relationship, education of close relationship, education of father, education of mother, job of close relationship, job of father, job of mother

Values are bolded if they achieved statistical significance at *p* ≤ 0.05

*** *p* < 0.001

### Relationship between PSQI and STAI/SDS

PSQI scores were significantly associated with adherence to STAI/SDS according to linear regression models before adjustment; after adjusting for age and gender (adjusted model 1); and after adjusting for age and gender as well as major, ethnicity, only child, birthplace, closest relationship, education of closest relationship, education of mother, education of father, job of closest relationship, job of mother and job of father (adjusted model 2) (*P* < 0.001) (Table 3).

**Table 3:** Linear regression analysis for PSQI associated with STAI and SDS in students, [β (95% CI)]

		STAI		SDS	
		β(95% CI)	*P* value	β(95% CI)	*P* value
PSQI	Unadjusted	2.51 (2.22,2.81)	<0.001	1.40 (1.21,1.59)	<0.001
	Adjusted 1	1.51 (1.16,1.17)	<0.001	0.88 (0.65,1.11)	<0.001
	Adjusted 2	1.48 (1.12,1.83)	<0.001	0.89 (0.66,1.12)	<0.001

*β* beta coefficient, *CI* confidence interval, *PSQI *Pittsburgh Sleep Quality Index, *STAI *State-Trait Anxiety Inventory, *SDS *Self-rating Depression Scale

Adjusted 1: Adjusted for gender, age

Adjusted 2: Adjusted for gender, age, major, ethnicity, only child, birthplace, closest relationship, education of closest relationship, education of mother, education of father, job of closest relationship, job of mother, job of father

### Relationship between STAI and SDS

STAI scores were significantly associated with SDS scores according to linear regression models before adjustment; after adjusting for age and gender (adjusted model 1); and after adjusting for age and gender as well as major, ethnicity, only child, birthplace, closest relationship, education of closest relationship, education of mother, education of father, job of closest relationship, job of mother and job of father (adjusted model 2) (*P* < 0.001) (Table 4).

**Table 4:** Linear aegression analysis for STAI associated with SDS in students, [β (95% CI)]

		SDS	
		β(95% CI)	*P* value
STAI	Unadjusted	0.49 (0.46,0.51)	<0.001
	Adjusted 1	0.48 (0.44,0.49)	<0.001
	Adjusted 2	0.48 (0.45,0.50)	<0.001

*β* beta coefficient, *CI* confidence interval, *SDS *Self-rating Depression Scale, *STAI *State-Trait Anxiety Inventory

Adjusted 1: Adjusted for gender, age

Adjusted 2: Adjusted for gender, age, major, ethnicity, only child, birthplace, closest relationship, education of closest relationship, education of mother, education of father, job of closest relationship, job of mother, job of father

### Mediation analysis

**Fig. 1:**
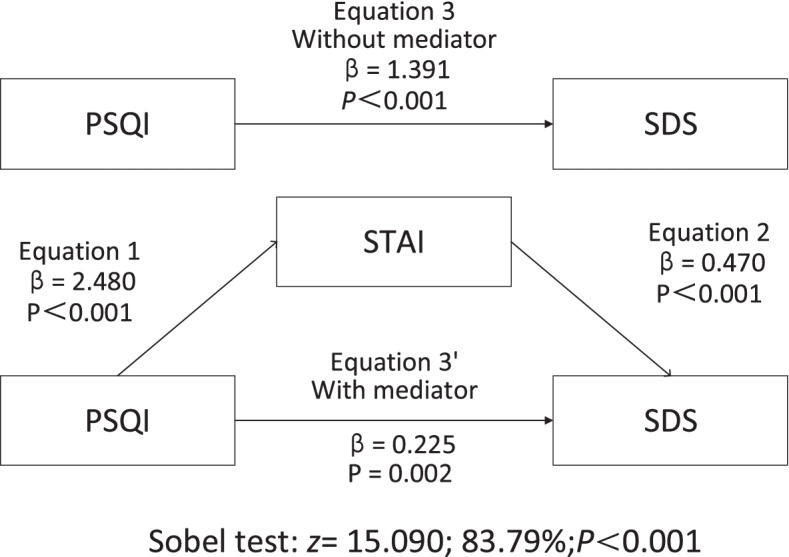
Mediation Analysis. Note: STAI = State-Trait Anxiety Inventory; PSQI = Pittsburgh Sleep Quality Index; SDS = Self-rating Depression Scale. The Sobel test was used to test the hypothesis that the indirect role was equal to 0, adjusting for potential confounders (gender, birthplace, grade, major, ethnicity, only child, close relationship, education of close relationship, education of father, education of mother, job of close relationship, job of father, job of mother). Values are bolded if they reached statistical significance at *p* ≤ 0.05

PSQI scores were positively associated with SDS scores among medical university students. Mediation analysis including the STAI revealed that the association between PSQI and SDS scores was mediated via STAI scores. STAI partially mediated the association between PSQI and SDS in this study, and this mediating variable accounted for 83.79% of the association when adjusting for potential confounders. PSQI scores were related to STAI (β = 2.480, *P* < 0.001) and SDS scores (β = 0.225, *P* < 0.001). STAI scores were also related to SDS scores (β = 0.470, *P* < 0.001). The final mediation models of the independent variable (PSQI), the mediating variable (STAI) and the dependent variable (SDS) are shown in Fig. 1.

### Subgroup analysis

Subgroup analyses of sex and birthplace are shown in Table 5. STAI partially mediated the association between PSQI scores and SDS scores in females (z = 10.313; indirect effect = 1.117, CI = 1.164 ~ 1.700, *P* < 0.001; direct effect = 0.315, CI = 1.976 ~ 2.818; *P* < 0.001) and rural students (z = 12.521; indirect effect = 1.213, CI = 1.222 ~ 1.699, *P* < 0.001; direct effect = 1.461, CI = 2.193 ~ 2.916; *P* < 0.001). STAI fully mediated the association between PSQI scores and SDS scores in males (z = 10.884; indirect effect = 1.217, CI = 1.161 ~ 1.722, *P* < 0.001; direct effect = 0.0149, CI = 1.980 ~ 2.661; *P* = 0.128) and urban students (z = 8.411; indirect effect = 1.104, CI = 1.213 ~ 1.687, *P* = 0.247; direct effect = 1.294, CI = 2.182 ~ 2.819; *P* < 0.001) (Table 5).

**Table 5:** Subgroup analysis of mediation models for SDS associated with PSQI mediated by STAI in medical university students

	Indirect effect	Direct effect	Total effect	Z	Sobel *p* value	Proportion of total effect that is mediated
Gender						
Male	1.217***	0.149	1.366***	10.884	<0.001	89.12
Female	1.117***	0.315**	1.432***	10.313	<0.001	78.01
Birthplace						
Urban	1.104***	0.189	1.294***	8.411	<0.001	85.37
Rural	1.213***	0.247**	1.461***	12.521	<0.001	83.07

*STAI* State-Trait Anxiety Inventory, *PSQI* Pittsburgh Sleep Quality Index, *SDS* Self-rating depression scale. Sobel-Goodman Mediation Test in adjusted models for gender, birthplace, grade, major, ethnicity, only child, close relationship, education of close relationship, education of father, education of mother, job of close relationship, job of father, job of mother. Values are bolded if they achieved statistical significance at *P* ≤ 0.05

** *p* < 0.01, *** *p* < 0.001

## Discussion

Previous studies have shown a bidirectional relationship between state-strait anxiety (depression symptoms) and sleep quality [36]. Our study confirmed that depression symptoms can be predicted by sleep quality, that higher levels of state-strait anxiety are a risk factor for depression symptoms and that state-strait anxiety can also be predicted by sleep quality. Moreover, our study further revealed that state-strait anxiety mediated the effect of sleep quality predicting on depression symptoms. In addition, the present study focused on the levels of sleep quality, state-strait anxiety, and depression symptoms among medical students during the COVID-19 pandemic and analyzed the variability of subgroups by gender and birthplace in this particular population.

The China National Mental Health Report (2019–2020) shows that university students have slightly higher rates of depression symptoms risk than adolescents nationally and adults nationally [37]. Among university students, medical majors have a higher prevalence rate of depression symptoms than nonmedical majors since medical students spend more time in university than students in other majors and face existential topics such as suffering and death [38]. In our study, 41.4% (518) of medical students reported suffering from depression symptoms. This figure is higher than the 28.9% combined prevalence rate for Chinese medical students that was reported in 2019 [4]. Chinese medical students also show a higher prevalence of depression symptoms than foreign medical students. The positive rates of depressive symptoms in medicine students reported in our study are higher than the 19.2% rate reported in a German study in 2018 [39] and higher than those of medical students in Middle Eastern countries (41.1%) who had the highest positive screening rate for depressive symptoms in a 2015 study across three countries [40]. These inconsistent findings may be related to the outbreak of the COVID-19 epidemic, during which medical students had more severe depressive symptoms [41].

Among the results of the characteristics of the respondents, we found a significant difference in sleep quality between men and women, which is similar to the results of previous studies [42], but found no difference in the distribution of depressive symptoms and anxiety state traits by gender, which is unlike other studies that reported a higher prevalence of depression symptoms and state-trait anxiety in women than in men [43, 44]. This may be related to the subject of our study. According to the Chinese educational system, the age of Chinese medical undergraduates is 18–23 years. More than half of the medical student population in our study was in their fourth year of college, which means that the average age was 22 years. Previous research examined the analysis of gender differences in depression in several countries around the world, including China, and noted that the significant decrease in gender differences in depressive symptoms from adolescence to the early 20s, while differences between the ages of 20–29 and later years were not significant [45].

Our findings showed that state-strait anxiety mediated the effect of sleep quality on depression. This is demonstrated by the fact that poor sleep quality causes the medial prefrontal cortex, which mediates the brain’s emotions, to be in a state of deactivation, which leads to increased anxiety [46]. Additionally, sleep deprivation also amplifies basic emotional responses and increases negative emotional states such as anxiety [47], and these emotional dysfunctions, such as anxiety, affect their normal interpersonal interactions [48]. Anxiety disorders eventually develop into depression symptoms due to interpersonal dysfunction [49].

Additionally, the results of the subgroup analysis further showed that STAI scores partially mediated the association between PSQI and SDS scores among females and rural students and fully mediated the association among males and urban students, which was also consistent with our study hypothesis. For the gender subgroup, poor sleep quality in women induces more complex mechanisms affecting depression, which may be due to the fact that women report both more intense positive and more intense negative emotions in their daily lives [50, 51]. For the birthplace subgroup, Chinese universities are established in cities, and there is a large gap between urban and rural areas in China. Moreover, there is a certain degree of migration discrimination and self-induced psychological distress for groups that come to live in urban from rural areas [39]. Therefore, for students who were born and had always lived in the city, the change in environment weakly impacts their emotions [37, 38, 40]. However, for those who were born in rural areas, university life was a huge change to the living environment, which could increase the complexity of emotional coping.

Several measures should be considered for improving both sleep quality and anxiety to relieve depression symptoms among university students. First, students should be encouraged to self-adjust through cognitive-behavioural therapy [52, 53] and comprehensive sleep management programs such as sleep hygiene education, relaxation training, and music therapy [54]. Second, consulting centres staffed with psychotherapists or trained counsellors should be established to provide professional psychological intervention and monitor students’ anxiety levels and psychological states [55]. Third, extracurricular activities should be enriched by offering certain physical exercises [56], courses such as tai chi [57, 58], yoga, and mindfulness training [59]. Moreover, medical students work an average of more than 90 hours per week during their inpatient rotations, sleep an average of 2 hours per night in an on-call status, and sleep an average of 4 hours less per night than they do at home [60], resulting in a significant reduction in sleep quality. Therefore, there is a need to make changes in policies regarding long shifts and working hours. Hospital administrators and policy-makers should limit shift work to 12–16 hours, schedule at least 10 hours of rest between shifts [61] and should not work more than 80 hours per week [62], thus ensuring quality sleep and more efficient work for medical students.

### Strengths and limitations

This study makes an important contribution to the literature by assessing the associations among the PSQI, STAI and SDS through data reported by medical students from four medical universities in Anhui Province. The present study confirms our research hypothesis that state-trait anxiety moderates the relationship between poor sleep quality and depression symptoms in university students. Additionally, we conducted subgroup analyses of gender and birthplace and showed that the extent to which poor sleep quality affects depression symptoms through state-trait anxiety was more pronounced in female medical students and in rural-born medical students. These findings all enrich the theory of depression symptoms-related research among medical students.

This article also has some limitations. First, our study is a cross-sectional study that can only account for the associations among the PSQI, STAI and SDS and cannot explain their causal relationship. Second, we used a convenience sampling method to collect questionnaire information from medical students within the four medical universities in Anhui Province, which may have affected the sample representativeness. However, we collected a sample of 1227 respondents, and this large sample was able to compensate for this shortcoming to some extent. Third, there was a possibility of recall bias, as all data were self-reported.

## Conclusions

In conclusion, this study confirms the importance of the association among depression symptoms, sleep quality, and state-trait anxiety. Sleep quality and state-trait anxiety have a significant predictive effect on depression symptoms. State-trait anxiety mediated the effect of sleep quality on depression symptoms, and a more complex mechanism was seen in rural and female medical students. These findings suggest that improving sleep quality and state-trait anxiety can meaningfully improve depression symptoms. Depression symptoms could be prevented and improved by encouraging students to self-adjust, providing professional psychological intervention and timely monitoring, enriching extracurricular activities, and taking changes in policies regarding long shifts and working hours to improve the quality of sleep and state characteristic anxiety in medical students.

## Supplementary Information

**Additional file 1: Supplementary Table 1.** Variable Description.

**Additional file 2: Supplementary Table 2.** The Selection Process of Covariates: Step 1 -Analyzing The Relationship Between the Covariate and Y (Y = SDS) One by One.

**Additional file 3: Supplementary Table 3.** The Selection Process of Covariates: Step 2 -Covariates Were Introduced into The Basic Model and Removed from The Complete Model to Observe the Change of the Regression Coefficient of X (X = PSQI-level).

**Additional file 4: Supplementary Table 4.** The Selection Process of Covariates: Step 2 -Covariates Were Introduced into The Basic Model and Removed from The Complete Model to Observe the Change of the Regression Coefficient of X (X = STAI score).

## Data Availability

All data generated or analyzed during this study are included in this published article [and its supplementary information files].

## Abbreviations

PSQI: Pittsburgh sleep quality index
S-AI: State anxiety
T-AI: Trait anxiety
STAI: State-Trait Anxiety Inventory
SDS: Depression Self-Rating Scale
