# Supplementary material for: Association of depression symptoms and sleep quality with state-trait anxiety in medical university students in Anhui Province, China: a mediation analysis
Source: BMC Med Educ. 2022 Aug 19;22:627. doi: 10.1186/s12909-022-03683-2 (PMC9388213; doi:10.1186/s12909-022-03683-2)
Supplement: Supplementary file 1 — Additional file 1: Supplementary Table 1. Variable Description. [file 12909_2022_3683_MOESM1_ESM.docx]

**Supplementary** **Table** **1** **Variable** **Description.**

| Variable name | Definition or code |
| --- | --- |
| Dependent variable |  |
| SDS-score | Score of Self-rating Depression Scale. |
| Mediation variable |  |
| STAI-score | Score of the State-Trait Anxiety Inventory |
| Independent variables |  |
| PSQI-score | Score of Pittsburgh Sleep Quality Index |
| Covariates |  |
| Age | -- |
| Gender | 1=Male; 2=Female. |
| Grade | 1=First year; 2=Second year; 3=Third year; 4=Fourth year; 5=Fifth year. |
| Major | 1=Medicine; 2=Other(Management, linguistics, law, etc.). |
| Ethnicity | 1=Han; 2=Minority. |
| Birthplace | Residence indicates the household living region and is defined by National Bureau of Statistics of the People's Republic of China. 1=Urban; 2=Rural. |
| Only child | 0=No; 1=Yes. |
| Close relationship | 1=Parents; 2=Grandparents; 3=Siblings; 4=Other(Friends, teachers, caregivers, etc.). |
| Education levels | Education level is a simplified version of 1997 International Standard Classification of Education (ISCED-97) codes. 1=Less than lower secondary education; 2=Upper secondary & vocational training; 3=Tertiary education. |
| Job | 1=Workers; 2=Farmers; 3=Civil servants, teachers and other intellectuals; 4=Businessmen; 5=Others |
