# Supplementary material for: Association of depression symptoms and sleep quality with state-trait anxiety in medical university students in Anhui Province, China: a mediation analysis
Source: BMC Med Educ. 2022 Aug 19;22:627. doi: 10.1186/s12909-022-03683-2 (PMC9388213; doi:10.1186/s12909-022-03683-2)
Supplement: Supplementary file 2 — Additional file 2: Supplementary Table 2. The Selection Process of Covariates: Step 1 -Analyzing The Relationship Between the Covariate and Y (Y = SDS) One by One. [file 12909_2022_3683_MOESM2_ESM.docx]

**Supplementary** **Table** **2** **The** **Selection** **Process** **of** **Covariates:** **Step** **1** **-**

**Analyzing** **The** **Relationship** **Between** **the** **Covariate** **and** **Y** **(Y=** **SDS)** **One** **by** **One.**

| Covariates | erm  t | beta Se. | 95%CI  Low | 95%CI Upp | *P*-value |
| --- | --- | --- | --- | --- | --- |
| Major  Ethnicity  Only child Birthplace Closest relationship  Education of closest  relationship Education of father Education of mother  Job of closest  relationship  Job of father  Job of mother | Non-medical  Minorities  Yes  Rural  Siblings  Grandfathers  Other  Elementary  Middle School  Elementary  Middle School  Elementary  Middle School  Farmer  Civil servants, teachers and other intellectuals Businessman Other  Farmer  Civil servants, teachers and other intellectuals Businessman Other  Farmer  Civil servants, teachers and other intellectuals Businessman Other | 1.6417  1.2942 1.8822 0.6379 0.6473 0.9597 1.5493 2. 1918  0.8066  0.7265 0.7604 0.7752 0.8011 0.9060  0.9303  0.8629  0.9849 0.9561 1.0545  0.8450  0.9000 1.0640 0.8681  0.9532  1.0145  0.9081  -0.0914  -0.3442  0.8087  1.8293  -0.0129  -0.5812  1.4547  1.4827  0. 1502  - 1.4349  0.4740  1.2186  -0. 1608  0.6434  - 1.9943  1.2974  0.7486  -0.5054  - 1.4680  0.0083  -0.5609  1. 1147  -0.8015  -0.4657 | -0.8950 -3.7806 - 1.5945 -0.4600 -0.0516 -3.0496 -4.8771  -0. 1262  0.0588 - 1.3402 -2.9542 - 1.0963 -0.5570  - 1.9843  - 1.0480  -3.9248 -0.5765 - 1.3183  -2. 1616  -3.2319 -2.0772 -2.2624  -0.7536  -2.7900  -2.2456 | 4. 1784 3.5978 0.9062 2.0773 3.7102 3.0237 3.7147  3.0357  2.9066 1.6405 0.0845 2.0442 2.9943  1.6627  2.3348  -0.0639 3. 1714 2.8155  1. 1508  0.2959 2.0937 1. 1407  2.9830  1. 1870  1.3143 | 0.2049  0.9613  0.5896  0.2118  0.0569  0.9933  0.7909  0.0716  0.0415  0.8435  0.0644  0.5542  0. 1788  0.8628  0.4561  0.0431  0. 1750  0.4779  0.5499  0. 1031  0.9938  0.5184  0.2425  0.4297  0.6082 |
