# Supplementary material for: Association of depression symptoms and sleep quality with state-trait anxiety in medical university students in Anhui Province, China: a mediation analysis
Source: BMC Med Educ. 2022 Aug 19;22:627. doi: 10.1186/s12909-022-03683-2 (PMC9388213; doi:10.1186/s12909-022-03683-2)
Supplement: Supplementary file 3 — Additional file 3: Supplementary Table 3. The Selection Process of Covariates: Step 2 -Covariates Were Introduced into The Basic Model and Removed from The Complete Model to Observe the Change of the Regression Coefficient of X (X = PSQI-level). [file 12909_2022_3683_MOESM3_ESM.docx]

**Supplementary** **Table** **3** **The** **Selection** **Process** **of** **Covariates:** **Step** **2** **-**

**Covariates** **Were** **Introduced** **into** **The** **Basic** **Model** **and** **Removed** **from** **The** **Complete** **Model** **to** **Observe** **the** **Change** **of** **the** **Regression** **Coefficient** **of** **X** **(X=** **PSQI-level).**

| Covariates | Basic model Complete model |  | Selected |
| --- | --- | --- | --- |
|  | Not bad Fair Good Not bad Fair | Good |  |
| Original coefficient  Major  Ethnicity  Only child  Birthplace  Closest relationship  Education of closest  relationship  Education of father Education of mother Job of closest relationship Job of father  Job of mother | - 1.0115 -7.5381 - 13.3707 -0.7183 -7. 1962  - 1.0554 -7.5366 - 13.3798 -0.6788 -7.2009  - 1.0160 -7.5432 - 13.3760 -0.7097 -7. 1853  -0.9214 -7.4616 - 13.2871 -0.6437 -7. 1404  -0.7472 * -7.3613 * - 13.2173 * -0.8306 -7.2196  -0.6780 * -7. 1205 * - 12.9634 * -0.8427 -7.2822  - 1. 1980 * -7.6941 * - 13.5065 * -0.8198 -7.2336  -0.7554 * -7.2778 * - 13.0774 * - 1.0777 -7.6611  - 1. 1906 * -7.7051 * - 13.5284 * -0.6792 -7. 1745  -2.0145 * -8.5436 * - 14.3192 * -0.7482 * -7.3566 *  - 1.5052 * -8. 1122 * - 13.9013 * -0.7039 -7. 1189  - 1.3710 * -7.9380 * - 13.7319 * - 1.2620 -7.8402 | - 12.8519 - 12.8530 - 12.8397 - 12.7817 - 12.8151 - 12.9356  - 12.9112  - 13.4152 - 12.8356 - 12.9997 * - 12.7931  - 13.5043 | Yes  Yes  Yes  Yes  Yes  Yes  Yes  Yes  Yes |

* Original coefficient changed more than 10%.
