# Supplementary material for: Association of depression symptoms and sleep quality with state-trait anxiety in medical university students in Anhui Province, China: a mediation analysis
Source: BMC Med Educ. 2022 Aug 19;22:627. doi: 10.1186/s12909-022-03683-2 (PMC9388213; doi:10.1186/s12909-022-03683-2)
Supplement: Supplementary file 4 — Additional file 4: Supplementary Table 4. The Selection Process of Covariates: Step 2 -Covariates Were Introduced into The Basic Model and Removed from The Complete Model to Observe the Change of the Regression Coefficient of X (X = STAI score). [file 12909_2022_3683_MOESM4_ESM.docx]

**Supplementary** **Table** **4** **The** **Selection** **Process** **of** **Covariates:** **Step** **2** **-**

**Covariates** **Were** **Introduced** **into** **The** **Basic** **Model** **and** **Removed** **from** **The** **Complete** **Model** **to** **Observe** **the** **Change** **of** **the** **Regression** **Coefficient** **of** **X** **(X=** **STAI** **score).**

| Covariates | Basic model | Complete model Selected |
| --- | --- | --- |
| Original coefficient  Major  Ethnicity  Only child  Birthplace  Closest relationship  Education of closest relationship  Education of father  Education of mother  Job of closest relationship  Job of father  Job of mother | 0.4857  0.4857  0.4861  0.4875  0.4855  0.4868  0.4849  0.4851  0.4855  0.4852  0.4857  0.4857 | 0.4851  0.4853  0.4848  0.4831  0.4854  0.4844  0.4856  0.4870  0.4849  0.4846  0.4845  0.4851 |

* Original coefficient changed more than 10%.
